# Tolosa-Hunt syndrome and recurrent painful ophthalmoplegic neuropathy, case reports: what to do and when?

**DOI:** 10.1186/s13052-023-01541-5

**Published:** 2023-11-27

**Authors:** Daniele Frattini, Alessandro Iodice, Carlotta Spagnoli, Susanna Rizzi, Carlo Alberto Cesaroni, Michela Cappella, Carlo Fusco

**Affiliations:** 1 Child Neurology Unit, Arcispedale Santa Maria Nuova Hospital - IRCCS, Viale Risorgimento 80, Reggio Emilia, 42123 Italy; 2 Child Neuropsychiatry Unit, S. Chiara Hospital, APSS, Largo Medaglie d’oro 9, Trento, 38122 Italy; 3 Pediatric Unit, Arcispedale Santa Maria Nuova Hospital - IRCCS, Viale Risorgimento 80, Arcispedale Santa Maria Nuova, Reggio Emilia, 42123 Italy

**Keywords:** Case report, Paediatric headache, Tolosa-Hunt syndrome, Ophthalmoplegic migraine, Recurrent painful ophthalmoplegic neuropathy

## Abstract

**Background:**

Tolosa-Hunt syndrome (THS) and recurrent painful ophthalmoplegic neuropathy (RPON) are rare diseases reported within the “Painful lesions of the cranial nerves” section of the International Classification of Headache Disorders-3^rd^ edition (ICHD-3). In case of a first painful attack, differential diagnosis could be challenging and many pitfalls are due to the rarity of the disorders and the lack of information about correct medical management in youngsters.

**Case presentation:**

Our main purpose was to report a new case of THS and a new case of RPON describing management and diagnostic investigation at the time of the first episode.

In both cases of THS (13 years old) and RPON (14 years old) a unilateral periorbital headache associated with acute onset of ipsilateral third cranial nerve paresis, scarcely responding to non-steroidal anti-inflammatory drugs (NSAID), was present at the beginning of the first attack. Brain MRI with "time-of-flight" (TOF) angiography and the need to administer steroids (after 72 h from onset) in order to stop pain were the most important handles allowing us to adopt the correct management both in THS or RPON since onset and to face recurrences in RPON by avoiding useless therapy during follow-up.

**Conclusion:**

Unilateral periorbital headache associated with third-fourth or sixth cranial nerve paresis should ideally be investigated with a full work-up, comprehensive of brain MRI with TOF angiography since the first attack. In cases with negative brain MRI spontaneous resolution should be considered and watchful waiting might be advisable before starting steroid therapy.

## Background

Tolosa-Hunt syndrome (THS) was first described by Tolosa and Hunt almost seventy years ago, but based on the literature search in the main medical databases, it was reported rarely during the paediatric age. Tolosa-Hunt syndrome (THS) is described as a very severe, unilateral periorbital headache associated with painful and restricted eye movements [1].

THS can be associated with painful ophthalmoplegia of one or more between the third, fourth, or sixth cranial nerve caused by granulomatous inflammation of unknown aetiology in the cavernous sinus, superior orbital fissure or orbital apex. Steroid therapy is recommended in THS although controversy exists regarding dosage, time and length of administration, independently from age [2].

Diagnosis is based on specific criteria by the International Classification of Headache Disorders revised last time in ICHD-3 beta in 2013 and not modified in the 2018 ICHD-3 [3, 4].

In all paediatric cases, conventional brain MRI demonstrates thickening of the cavernous sinus, superior orbital fissure and/or orbital apex with increased contrast enhancement after gadolinium administration, which is not always detectable at the onset of painful ophthalmoplegia and might be only recognized after several weeks [5].

Ophthalmoplegic migraine, renamed "Recurrent Painful Ophthalmoplegic Neuropathy" (RPON) in the ICHD-3, is a rare neurologic disorder characterized by recurrent attacks of ophthalmoplegia in association with ipsilateral headache [3, 4]. According to the ICHD criteria, RPON can be considered after at least two attacks of a migraine-like headache, associated with paresis of the ocular cranial nerves occurring within 4 days since symptoms onset [4].

In a recent paper, Falsaperla and co-workers suggested to consider a diagnosis of recurrent painful ophthalmoplegic neuropathy even at the first attack, provided that the typical MRI pattern with thickening of the involved cranial nerve and reduced post-contrast enhancement are detected [6].

We report on a new case of THS and a new case of RPON, and describe management and diagnostic investigations undertaken at the first ever episode.

## Case presentation

### Patient 1

We report the case of a 13-year-old female referred to our hospital due to persistent headache in the right periorbital and right frontal area, associated with vomiting, which was preceded (one month before) by SARS-COV2 paucisymptomatic infection and matched after two days by dizziness, right gaze diplopia and right upper eyelid oedema. Neither fever nor other clinical neurological findings were evident. The patient was born at term from healthy unrelated parents and had normal neurodevelopmental achievements.

On the second day aince the onset, neurologic examination showed slight outward and downward moving of the right eye only to the middle when looking inward, right ptosis and a mydriatic pupil confirming a third cranial nerve palsy.

At the endocrinological examination, a height of 148.2 cm (3rd centile), a weight of 73.75 kg (> 97th centile) and a BMI of 33.58 kg/m^2^ were found. Abdominal ultrasound documented hepatic steatosis. Blood tests showed LDL cholesterol values of 129 mg/dL (normal values < 116) and HDL cholesterol of 31 mg/dL (normal values > 45), remaining values within normal limits. BrainCT scan was normal; brain MRI including TOF angiography (3d-TOF and phase-contrast) showed linear thickening of the right tentorial profile, with intense contrast enhancement, in the whole segment starting from the anterior insertion and thickening of the lateral wall of the cavernous sinus, better appreciated in the T1-weighted and increased TR time images.

**Fig. 1:**
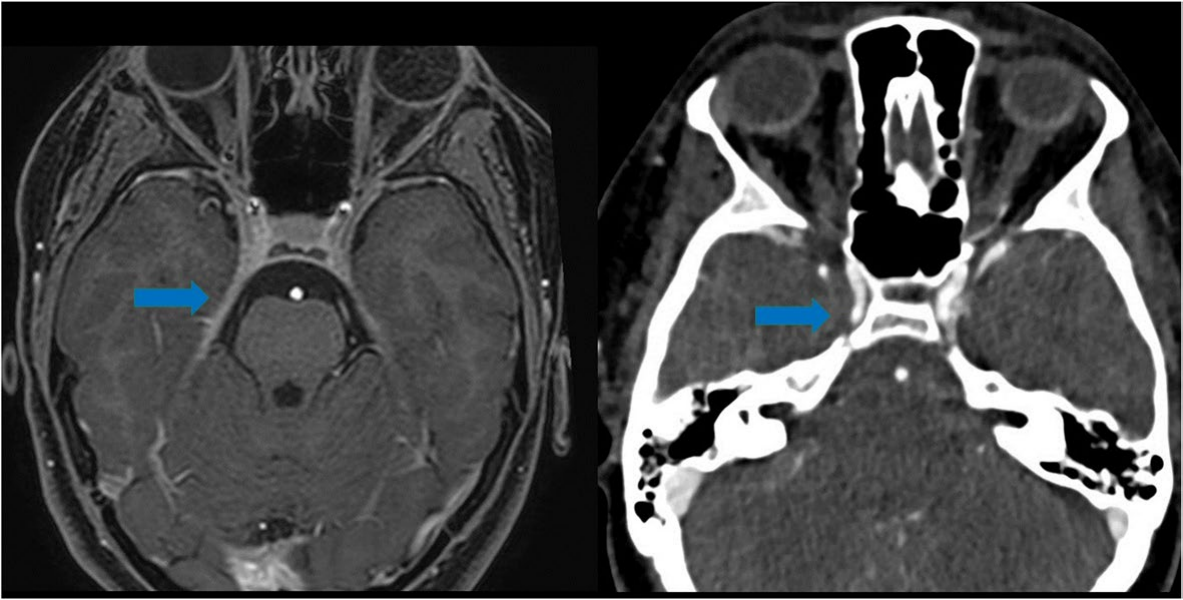
Brain MRI (left, axial T1-weighted with gadolinium) showed linear thickening of the right tentorial profile (blue arrow) in the whole segment starting from the anterior insertion and concomitant thickening of the lateral wall of the cavernous sinus. CT angiography (right) confirmed abnormal blood vessel thickening in the same region (blue arrow)

CT angiography confirmed abnormal blood vessel thickening in the same region of the right tentorial profile and at the lateral wall of the cavernous sinus (Fig. 1). Although with a clinical and neuroradiological suspicion of THS, alternative inflammatory and infective diseases were also investigated with a comprehensive blood work-up, particularly investigating viral and bacterial agents, thyroid dysfunction and antiganglioside antibodies. Cerebrospinal fluid analysis (CSF), including antiganglioside antibodies, was unremarkable.

After a preliminary diagnosis of THS, treatment was started. Steroids therapy with intravenous metiprednisolone (1 g/day) for five days dramatically improved frontal headache and third cranial nerve plasy. Metilprednisolone therapy was followed by oral deflazacort for 3 months (0.9 mg/kg per day). The patient fully recovered after 3 months of treatment. To date, no relapse has occurred. With respect to MRI findings, clinical features at presentation and follow-up, the diagnosis of THS was then confirmed (Table 1).

**Table 1:** Diagnostic criteria for Tolosa-Hunt Syndrome and Recurrent Painful and Ophthalmologic Neuropathy according to ICHD-3

	**Tolosa-Hunt syndrome**	**Recurrent painful ophthalmoplegic neuropathy**
DESCRIPTION	Unilateral orbital or periorbital pain associated with paresis of one or more between the 3^rd^, 4^th^ and/or 6^th^ cranial nerves caused by a granulomatous inflammation in the cavernous sinus, superior orbital fissure or orbit	Repeated attacks of paresis of one or more ocular cranial nerves (commonly the 3^rd^), with ipsilateral headache
DIAGNOSTIC CRITERIA	A. Unilateral orbital or periorbital headache fulfilling criterion C	A. At least two attacks fulfilling criterion B
		B. Both of the following:
	B. Both of the following:	
	1. granulomatous inflammation of the cavernous sinus, superior orbital fissure or orbit, demonstrated by MRI or biopsy	1. unilateral headache
		2. ipsilateral paresis of one, two or all three ocular motor nerves
	2. paresis of one or more of the ipsilateral the 3^rd^, 4^th^ and/or 6^th^ cranial nerves	
		C. Orbital, parasellar or posterior fossa lesion has been excluded by appropriate investigation
	C. Evidence of causation demonstrated by both of the following:	D. Not better accounted for by another ICHD-3 diagnosis
	1. headache is ipsilateral to the granulomatous inflammation	
	2. headache has preceded paresis of the the 3^rd^, 4^th^ and/or 6^th^ nerves by ≤ 2 weeks, or developed with it	
	D. Not better accounted for by another ICHD-3 diagnosis	
COMMENTS	Some reported cases of Tolosa-Hunt syndrome had additional involvement of the 5^th^ nerve (commonly the first division) or optic, 7^th^ or 8^th^ nerves. Sympathetic innervation of the pupil is occasionally affected	Some data suggest that headache can develop up to 14 days prior to ocular motor paresis
		Gadolinium enhancement or nerve thickening can be demonstrated using MRI
	Careful follow-up is required to exclude other causes of painful ophthalmoplegia such as tumours, vasculitis, basal meningitis, sarcoid or diabetes mellitus	
	Pain and paresis of Tolosa-Hunt syndrome resolve when adequately treated with corticosteroids	

### Patient 2

We report on the case of a 10-year-old female presenting with fronto-orbital headache on the left side with gradual onset of mild eyelid ptosis and diplopia due to divergent strabismus with ocular convergence deficit and limitation of inward and upward movements of the left eye. Three hours after headache had begun, a partial third cranial nerve palsy was confirmed at the neurological examination. No other clinical or neurological findings, except for eye discomfort when exposed to bright light, were found. Family history was unremarkable.

A brain CT scan was normal. Non-steroidal anti-inflammatory drugs (NSAID) were used as first-line therapy (iv paracetamol 15 mg/kg). Headache transiently ceased within two hours without third cranial nerve regression.

Brain MRI comprehensive of TOF angiography (3d-TOF and phase-contrast) performed 24 h after symptoms onset was completely normal. No contrast enhancement was documented. CSF and blood tests excluded inflammatory diseases. Due to the partial clinical improvement and negative brain MRI, a preliminary diagnosis of RPON was made and treatment with ibuprofene 10 mg/kg/per dose, three times per day was started.

Headache and third cranial nerve palsy completely recovered within 48 h from onset.

Six months later, a second attack with the same features occurred. During a three years’ follow-up, the presented repeated attacks of third cranial nerve palsy with ipsilateral headache treated with NSAID therapy, lasting approximately 4–72 h. According to the ICSD-3 criteria, a diagnosis of RPON was formulated (Table 1).

## Discussion and conclusion

THS and RPON in children are rare causes of painful ophthalmoplegia. At onset, a correct diagnosis is challenging since the differential diagnosis include neoplasm (e.g. schwannomas), aneurysm, carotid dissection, temporal arteritis, sarcoidosis, and infectious etiologies.

As confirmed by a recent review, 12 cases of THS were reported in the past 10 years [5, 7], while RPON seems to be more common in the paediatric age, even if this diagnosis should be confirmed after an adequate follow-up period [8]. We compared our patients’ characteristics with previously described patients, with respect to MRI findings in the acute phase, response to therapy, symptoms and duration.

In clinical practice, brain MRI with or without intravenous administration of paramagnetic fluid is routinely performed in the acute phase, but sometimes there is a time delay from the onset and TOF angiography is not included [5]. In both our patients, brain MRI was performed within 72 h from the onset and the presence of abnormal blood vessel thickening of the cavernous sinus led to prompt THS diagnosis.

In paediatric patients with THS, the main site of involvement is the cavernous sinus [9]. Granulomatous inflammation, even affecting the superior orbital fissure or the orbit could be detected only by angiographic methods or biopsy. On the other hand, in almost 60% of RPON cases in patients younger than18 years of age, brain MRI reveals asymmetric thickening or gadolinium enhancement of the cisternal segment of the affected cranial nerve [9, 10].

In RPON a second attack with unilateral headache and ipsilateral paresis is needed to confirm the diagnosis, but negative brain MRI with TOF angiography easily excludes orbital, parasellar or posterior fossa lesions, avoiding inadequate therapy. In our cases, we considered RPON since the beginning due to spontaneous resolution and negative brain MRI, even if the diagnosis was confirmed thanks to the recurrence of additional attacks during follow-up. Moreover, follow-up can be required not only to confirm a diagnosis of RPON, but even in the case of misdiagnosed nerve schwannomas [11].

According to ICHD criteria, in cases with negative brain MRI or cranial nerve thickening and gadolinium enhancement, the diagnosis of RPON could only be suspected and not confirmed before a second episode, whereas a diagnosis of THS could be made since the first attack.

Some controversies arise regarding which cases should be eligible for steroid therapy, and when [5].

Steroid therapy, although effective and recognized as the first-line therapy in THS, should be introduced after extensive investigations confirming granulomatous inflammation or cranial nerve swelling. It was successful in all reported paediatric cases of THS, except for one with ophthalmoplegia and granulomatous inflammation of the cavernous sinus, but without pain and spontaneous resolution [6, 12]. Conversely, more than half of the cases (both adults and younger) with RPON completely recovered within 72 h without specific treatment [9].

In case of negative brain MRI with good response to NSAID therapy, it would be preferable to adopt a waiting conduct, with a timeline depending on clinical features and a cut-off of 72 h from the onset [8]. On the other hand, corticosteroids could be useful in the acute case of RPON when nerve inflammation is documented [5, 9]. As performed in our patients, lumbar puncture with cytological CSF examination is mandatory even in case of a negative brain MRI, in order to exclude hematologic neoplastic diseases primarily affecting the central nervous system. A lumbar puncture should be possibly performed within 48 h from symptoms onset and before steroid treatment.

**Fig. 2:**
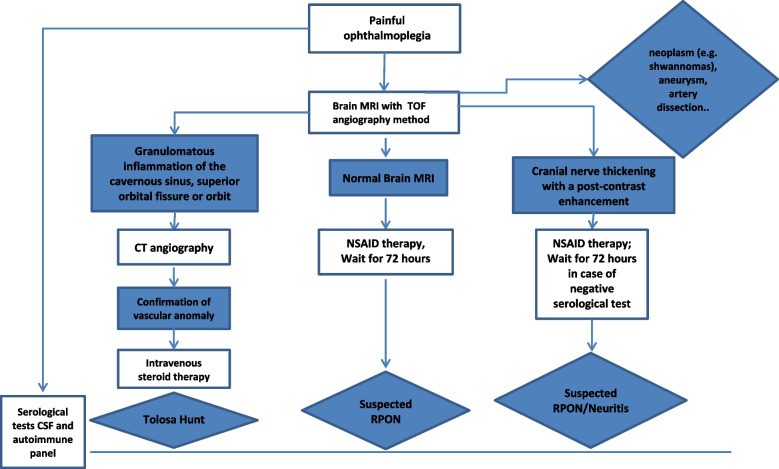
Diagnostic and therapeutic algorithm. MRI: Magnetic Resonance Imaging; CT: Computer Tomography; CSF: cerebrospinal fluid; NSAID: non-steroidal anti-inflammatory drugs; RPON: recurrent painful ophthalmoplegic neuropathy

In relapsing cases with prompt resolution after steroids therapy, an inflammatory mechanism with cranial nerve neuritis should be always investigated, with particular attention to atypical presentation of anti-GQ1b antibody syndrome [13, 14]. In cases of confirmed THS or suspected RPON, without spontaneous resolution, we propose the treatment strategy previously adopted in our units for neuroimmune disorders: 25 mg/kg/day (or 1 g/day, for patients over 40 kg) iv methyl-prednisolone for 5 days, followed by oral deflazacort 0.9 mg/kg/day for 1–3 months depending on the clinical and neuroradiological evolution [15]. Our diagnostic and therapeutic algorithm (Fig. 2) can support clinical diagnosis and correct treatment of unilateral periorbital headache associated with third-fourth or sixth cranial nerve paresis in children.

## Data Availability

All data generated or analysed during this study are included in this published article.
